# *FBXW7*-loss Sensitizes Cells to ATR Inhibition Through Induced Mitotic Catastrophe

**DOI:** 10.1158/2767-9764.CRC-23-0306

**Published:** 2023-12-21

**Authors:** Siobhan O'Brien, Tajinder Ubhi, Lucie Wolf, Krishna Gandhi, Sichun Lin, Naz Chaudary, Neesha C. Dhani, Michael Milosevic, Grant W. Brown, Stephane Angers

**Affiliations:** 1 Department of Biochemistry, University of Toronto, Ontario, Canada.; 2 Terrence Donnelly Centre for Cellular and Biomolecular Research, Toronto, Ontario, Canada.; 3 Princess Margaret Cancer Centre, Toronto, Ontario, Canada.; 4 Ontario Cancer Institute, Toronto, Ontario, Canada.; 5 Department of Radiation Oncology, University of Toronto, Ontario, Canada.; 6 Institute of Medical Science, University of Toronto, Ontario, Canada.; 7 Leslie Dan Faculty of Pharmacy, University of Toronto, Ontario, Canada.

## Abstract

*FBXW7* is a commonly mutated tumor suppressor gene that functions to regulate numerous oncogenes involved in cell-cycle regulation. Genome-wide CRISPR fitness screens identified a signature of DNA repair and DNA damage response genes as required for the growth of *FBXW7*-knockout cells. Guided by these findings, we show that *FBXW7*-mutant cells have high levels of replication stress, which results in a genotype-specific vulnerability to inhibition of the ATR signaling pathway, as these mutant cells become heavily reliant on a robust S–G_2_ checkpoint. ATR inhibition induces an accelerated S-phase, leading to mitotic catastrophe and cell death caused by the high replication stress present in *FBXW7^−^^/^^−^* cells. In addition, we provide evidence in cell and organoid studies, and mining of publicly available high-throughput drug screening efforts, that this genotype-specific vulnerability extends to multiple types of cancer, providing a rational means of identifying responsive patients for targeted therapy.

**Significance:**

We have elucidated the synthetic lethal interactions between *FBXW7* mutation and DNA damage response genes, and highlighted the potential of ATR inhibitors as targeted therapies for cancers harboring *FBXW7* alterations.

## Introduction

F-box and WD-repeat containing protein 7 (FBXW7) is a substrate recognition component of a Skp1-Cul1-Fbox (SCF) E3 ligase complex. *FBXW7* is a tumor suppressor gene with mutations and deletions present across a wide range of cancers, most commonly in cervical, uterine, and colorectal tumors. FBXW7 recognizes several cellular proto-oncogenes for ubiquitination and subsequent degradation. Some of these substrates have been implicated in oncogene-induced replication stress, including cyclin E and c-Myc (1, 2). FBXW7 itself has been implicated in the response to DNA damage through its interaction with and degradation of DNA damage response proteins such as PLK1, BLM, and SOX9 (3–5). In addition, FBXW7 plays a direct role at sites of DNA damage through ubiquitination and subsequent activation of XRCC4, a factor required for non–homologous end joining (6). Considering that high levels of DNA damage and replication stress are hallmarks of cancer, targeting the replication stress response is an active area of therapeutic development. There are numerous small molecule drugs that target DNA damage and repair pathways that are in various stages of clinical trials, and some, like the PARP inhibitor Olaparib, have been approved for treatment of patients with cancer (7). One set of inhibitors that are currently undergoing clinical trials with positive early data are inhibitors of the ATR kinase, the core kinase that drives the cellular response to single-stranded DNA (ssDNA) accumulation in cells—a hallmark of replication stress. AZD6738 is an ATR inhibitor that prevents the activation of the ssDNA-RPA-ATR-CHK1 signaling cascade (8, 9). Inhibition of ATR induces an early S-phase exit, with accumulation of G_2_–M factors occurring during active DNA replication (8, 9), which leads to replication stress and mitotic catastrophe. AZD6738 has shown promising results in phase I clinical trials targeting refractory solid tumors (10) both as a single agent and in combination with other standard-of-care therapeutics. To date, little work has been done to identify patient subsets that may respond more favorably to inhibition of ATR signaling outside of core DNA damage repair mutations found in some cancers. Considering the acceptable safety and efficacy profiles of ATR inhibitors in early-stage clinical trials, understanding which patients may be most responsive to this drug is an important step to maximize its clinical success as a targeted therapy.

Here, using genome-wide isogenic CRISPR fitness screens in an engineered cell line that requires *FBXW7*-loss for survival (11), we uncovered synthetic lethal interactions between *FBXW7* mutation and DNA damage response genes. We uncovered that *FBXW7^−^^/^^−^* cells and organoids harbor high levels of replication stress creating a therapeutic vulnerability that can be exploited with ATR inhibitors. Specifically, we have uncovered that the high levels of replication stress in *FBXW7^−^^/^^−^* cells makes them reliant on the S–G_2_ checkpoint and that ATR inhibition triggers mitotic catastrophe. Important, our work presents evidence demonstrating that presence of *FBXW7* mutations could represent a biomarker directing the use of ATR inhibitors.

## Materials and Methods

### Cell Culture and Lentivirus Production

HPAF-II (RRID:CVCL_0313), HEK293T (RRID:CVCL_0063), C33A (RRID:CVCL_1094), and SiHa (RRID:CVCL_0032) cells were grown in DMEM + 10% FBS (Gibco) and 1% antibiotic and antimycotic (Gibco), Caski (RRID:CVCL_1100; ATCC) cells were cultured in RPMI (Gibco) + 10% FBS and 1% antibiotic and antimycotic, all at 37°C and 5% CO_2_ and 95%–100% humidity. Cells were routinely tested for *Mycoplasma* (Lonza) every 4–6 months. For lentivirus production, HEK293T cells were seeded to 60% confluence, and the following day transfected with 6 µg target plasmid (see Table 1 for oligo list), 6 µg pSPAX (Addgene #12260), and 1 µg pMD2.G (Addgene #12259) in 60 µg polyethylenimine (Sigma-Aldrich) and Opti-MEM (Gibco). A total of 24 hours posttransfection, media was replaced. Lentivirus was harvested 48 hours posttransfection, filtered through a 0.45 µm filter, and aliquoted and stored at −80°C prior to use. All cell lines were authenticated by short tandem repeat analysis at The Center for Applied Genomics (TCAG), Toronto, ON. Cell lines were used for 25–30 doublings before thawing fresh. Dose–response assays were seeded at 5,000 cells/well in a 96-well plate, treated with corresponding compounds the following day, and incubated for 5–7 days as indicated. Cell viability was read out using Cell Titer Glo (Promega) and luminescence values were read out on an Envision MultiLabel plate reader. IC_50_ calculations were performed in GraphPad Prism (RRID SCR_002798). All small molecule compounds were purchased from Selleck Chemicals.

**TABLE 1:** Oligo list

Oligo	Sequence
sgFBXW7br	ACAGAATTGATACTAACTGG
sgFBXW7br_TIDE_F	GGGATTGATGAACCATTGCACA
sgFBXW7br_TIDE_R	GCATTATTTTTCCTGGCTGACGAA
sgAAVS1	GTCCCCTCCACCCCACAGTG
sgAAVS1_TIDE_F	TGTCATGGCATCTTCCAGGG
sgAAVS1_TIDE_R	GTCTGAAGAGCAGAGCCAGG
sgPPP2CA-1	TACAGCTCACCTTCTCGCAG
sgPPP2CA-2	ATGGGAGATTATGTTGACAG
sgCDC25B-1	CGCCCGTGCAGAATAAGCG
sgCDC25B-2	GGCACTTGCTGTACATGACG
sgCCNE1-1	AGCCAGGACACAATAGTCAG
sgCCNE1-2	CCAAAATCGACAGGACGGCG

### Genome-wide Screen

HPAF-II wild-type and *FBXW7^−^^/^^−^* isogenic cells (11) were infected with the Toronto knockout library version 3 (TKOv3, Addgene #90294)—a pooled single-guide RNA lentiviral library (12) at a multiplicity of infection (MOI) of 0.3, in the presence of 8 µg/mL polybrene (Sigma-Aldrich) for 24 hours. Cells were treated with 2 µg/mL puromycin (Thermo Fisher Scientific) for 48 hours. Following selection, pooled cells were split into three replicates, and passed every 4 days for 24 days, maintaining 18 million cells per replicate. Cell pellets at T = 0, 12, and 24 days were collected, and genomic DNA extracted using the QIAmp DNA Blood Maxi Kit (Qiagen). Genomic DNA samples were amplified and barcoded using i5 and i7 adaptor primers for Illumina next-generation sequencing. Barcoded PCRs were sequenced with the Illumina HiSeq2500 with read depths of 200-fold coverage. Sequenced guide RNAs (gRNA) were mapped to the TKOv3 library using MAGeCK 0.5.3 (13). Read counts were normalized and fold change of gRNA distribution compared with T = 0 was calculated using the BAGEL package (14). BAGEL analysis was performed, and Bayes factors were compared between HPAF-II wildtype and isogenic cell lines. Z-scores of differential Bayes Factors between wild-type and isogenic cell lines were calculated.

### Chemogenomic Screen

HPAF-II *FBXW7^−^^/^^−^* cells were infected with the TKOv3 genome-wide lentiviral CRISPR-Cas9 library at a MOI of 0.3 in the presence of 8 µg/mL polybrene. The following day, media was replaced with full media containing 2 µg/mL puromycin. Cells were left to select for 2 days. Following selection, cells were collected, counted, and seeded at 18 million cells per replicate—two replicates for the untreated arm and two replicates for the LD50 arm. The following day, LD50 cells were treated with 100 nmol/L AZD6738. Cells were maintained in culture for approximately 26 days. Cell pellets at T = 0, 12, and 26 days were collected, and genomic DNA extracted using the QIAmp DNA Blood Maxi Kit (Qiagen). Genomic DNA samples were amplified and barcoded using i5 and i7 adaptor primers for Illumina next-generation sequencing. Barcoded PCRs were sequenced with the Illumina HiSeq2500 with read depths of 200-fold coverage. Sequenced gRNAs were mapped to the TKOv3 library using MaGECK 0.5.3 (13). Read counts were normalized, and Z-scores calculated using DrugZ (15).

### Western Blotting

All samples were lysed in 4X Laemmli Sample Buffer (50 mmol/L Tris-HCl pH 6.8, 2% SDS, 10% glycerol, 1% β-mercaptoethanol, 12.5 mmol/L Ethylenediaminetetraacetic acid (EDTA), 0.02% bromophenol blue). Lysates were sonicated, boiled, and centrifuged to pellet insoluble material. Approximately 10 µg of protein was loaded per sample on a 4%–15% SDS-PAGE Stain-Free TGX precast gel (Bio-Rad). Gels were run at 150 V for approximately 60 minutes. Gels were transferred to methanol activated polyvinylidene difluoride (Bio-Rad) at 90 V for 120 minutes. Membranes were blocked in 5% milk in TBS (pH 7.4) + 1% Tween-20 (TBS-T) for 1 hour and incubated with corresponding primary antibodies overnight (see Table 2 for antibody list). The following day, membranes were washed four times in TBS-T, and incubated with corresponding secondary antibodies for 1 hour, in 5% milk in TBS-T, at room temperature with agitation. Membranes were washed and detected using SuperSignal West Pico PLUS chemiluminescent substrate (Thermo Fisher Scientific) and imaged on the Chemidoc-MP (Bio-Rad).

**TABLE 2:** Antibody list

Target	Vendor	Catalog no.	RRID
pCHK1 (S345)	Cell Signaling Technology	2348S	AB_331212
CHK1	Cell Signaling Technology	2360S	AB_2080320
53BP1	BD Biosciences	612522	AB_2206766
gH2AX	Cell Signaling Technology	9718T	AB_2118009
RPA32	Abcam	ab2175	AB_302873
FBXW7	Bethyl Laboratories	A301-720A	AB_1210897
GAPDH	Thermo Fisher Scientific	AM4300	AB_2536381
CDC25B	Thermo Fisher Scientific	PA583441	AB_2790596
PP2A-C	Cell Signaling Technology	2038	AB_2169495
Cyclin E	Thermo Fisher Scientific	321600	AB_2533067
Ki67	Thermo Fisher Scientific	PA519462	AB_10981523
EpCAM-APC	Miltenyi Biotec	130-111-000	AB_2657497
pH3-S10	Cell Signaling Technology	9701	AB_331535
Cyclin B1	BD Biosciences	554176	AB_395287
BrdU	Abcam	ab6326	AB_305426
Anti-BrdU clone B44	BD Biosciences	347580	AB_10015219
Anti-ssDNA	Millipore	MAB3034	AB_94645

### Confocal Microscopy

Cells were grown on 22 × 22 mm coverslips (VWR) overnight. Following any treatments, the cells were harvested as follows—dependent on staining.

#### RPA32 Foci Staining

Cells were washed with ice-cold PBS and incubated for 10 minutes in nuclear extraction buffer (20 mmol/L HEPES pH7.5, 20 mmol/L NaCl, 5 mmol/L MgCl_2_, 1 mmol/L dithiothreitol (DTT), 0.5% NP-40, protease inhibitor cocktail) for 10 minutes at 4°C. Extraction buffer was washed from cells, and cells were fixed in 2% paraformaldehyde (PFA; Electron Microscopy Sciences) for 10 minutes at room temperature. Coverslips were blocked in blocking buffer (10% goat serum, 0.5% NP-40, 0.5% saponin in PBS) for 30 minutes at room temperature. Fixed coverslips were incubated with a 1:500 dilution of mouse anti-RPA32 antibody (Abcam) overnight at 4°C. The following day, coverslips were washed 3X in ice-cold blocking buffer, and a final incubation for 1 hour at room temperature with anti-Mouse AlexaFluor-488 (Thermo Fisher Scientific) secondary antibody. Following washing, coverslips were mounted to slides using ProLong Gold + DAPI (Thermo Fisher Scientific) mounting media.

#### 53BP1 and EdU Staining

One hour before harvest, cells were pulsed with 10 µmol/L EdU. Following treatment, coverslips were washed in PBS, and then fixed and permeabilized in HTEMF buffer (20 mmol/L HEPES pH 6.8, 10 mmol/L EDTA, 0.2% Triton X-100, 1 mmol/L MgCl_2_, and 4% PFA) for 20 minutes at room temperature. Cells were then washed and blocked in 3% BSA in PBS-T (PBS + 0.5% Triton X-100) for 1 hour at room temperature. Click-IT cocktail was made as per manufacturer's instructions (Thermo Fisher Scientific). Following block, coverslips were incubated in Click-IT reaction buffer at room temperature for 1 hour, in the dark. Coverslips were then washed and incubated overnight with 1:1,000 dilution of mouse anti-53BP1 antibody at 4°C. The following day, coverslips were washed 3X in ice-cold blocking buffer, and a final incubation for 1 hour at room temperature with anti-Mouse AlexaFluor-488 (Thermo Fisher Scientific) secondary antibody. Following washing, coverslips were mounted to slides using ProLong Gold + DAPI (Thermo Fisher Scientific) mounting media.

#### γH2AX Staining

Coverslips were harvested through fixation in 4% PFA in PBS for 15 minutes at room temperature. Cells were permeabilized using 0.25% Triton X-100, blocked in 5% BSA in PBS, and incubated overnight with primary antibody. The following day, coverslips were washed 3X in ice-cold blocking buffer, and a final incubation for 1 hour at room temperature with anti-Mouse AlexaFluor-488 (Thermo Fisher Scientific) secondary antibody. Following washing, coverslips were mounted to slides using ProLong Gold + DAPI (Thermo Fisher Scientific) mounting media.

#### pH3-S10 Staining

Coverslips were harvested through fixation in 4% PFA in PBS for 15 minutes at room temperature. Cells were permeabilized using 0.25% Triton X-100, blocked in 5% BSA in PBS, and incubated overnight with primary antibody. The following day, coverslips were washed 3X in ice-cold blocking buffer, and a final incubation for 1 hour at room temperature with anti-Rabbit AlexaFluor-488 (Thermo Fisher Scientific) secondary antibody. Following washing, coverslips were mounted to slides using ProLong Gold + DAPI (Thermo Fisher Scientific) mounting media. Images were collected at 20X magnification. Anaphase bridge images were collected at 63X magnification.

#### Image Acquisition and Analysis

Images were acquired at 40X (unless indicated) on a laser scanning confocal microscope (LSM700, Carl Zeiss) at 8-bit with Plan-Apochromat 40X/1.4NA oil immersion objective using Zen software. Five images per treatment per replicate were collected. Foci were counted using a CellProfiler (Broad Institute).

### PIP-FUCCI Reporter Incucyte Assay

HPAF-II wild-type and *FBXW7^−^^/^^−^* cells PIP-FUCCI expressing cells, described in ref. 11, were treated with varying doses of AZD6738 and mitotic accumulation was tracked in the Incucyte S3 (Sartorius IncuCyte S3 Live Cell Analysis System, RRID: SCR_023147) at 20X magnification for 48 hours. Mitotic cells were measured by area, marked as GFP and mCherry double positive, and normalized to untreated conditions.

### EdU Flow Cytometry

HPAF-II wild-type and *FBXW7^−^^/^^−^* cells were grown in the logarithmic growth phase. Two hours before harvest, cells were pulsed with 10 µmol/L EdU. Cells were harvested with trypsin, washed extensively, and fixed in Click-IT fixative (Thermo Fisher Scientific) for 15 minutes at room temperature. Cells were then washed and resuspended in Click-IT permeabilization buffer for 15 minutes at room temperature. Next, click reaction was performed in Click-IT staining solution (Thermo Fisher Scientific) as per manufacturer's instructions, for 30 minutes in the dark. Cells were then washed and resuspended in permeabilization buffer with 20µg/mL propidium iodide (BioShop) and 100 µg/mL RNAse A (Thermo Fisher Scientific). Cells were filtered through a 4 µm mesh filter and incubated at room temperature for 20 minutes before acquisition on a Beckman Coulter CytoFLEX flow cytometer. Cells were gated for singlets, and cell-cycle phase was determined using the intensity of propidium iodide in the PE channel and FITC channel.

### Organoid Isolation, Culture, Drug Treatment, and Imaging

#### Isolation

Patient-derived cervical tumor xenograft tissue was excised from a mouse and stored in DMEM-F12 (Thermo Fisher Scientific) on ice until processing. Upon receipt, tumor was mechanically dissociated using surgical scissors, taking care to remove any fat or blood vessels. Tumor pieces were washed 6–8× in Extreme DMEM-F12 (Wisent) + 1X antibiotic-antimycotic (Thermo Fisher Scientific) + 2 mmol/L HEPES + 2 mmol/L GlutaMAX-I, hereby referred to as DMEM+++). Tumor pieces were then incubated in 0.125 mg/mL collagenase (Sigma-Aldrich) and 0.125 mg/mL dispase (Sigma-Aldrich in DMEM+++ for 2–2.5 hours, shaking at 220 rpm at 37°C. Following enzymatic dissociation, tumor pieces were washed 6–8x in DMEM+++ and filtered through a 100 µm mesh filter. Tumor was then collected and resuspended in reduced growth factor Matrigel (Corning) and plated as 25 µL domes in 48-well plates. Organoids were cultured in cervical organoid media (DMEM+++, 1X B27, 2.5 mmol/L nicotinamide, 1.25 mmol/L n-Acetylcystein, 10 µmol/L Y27632, 0.5 µmol/L A8301, 10 µmol/L forskolin, 100 ng/mL FGF-10, 25 ng/mL FGF-7, 100 ng/mL noggin, 1 µmol/L SB202190; ref. 16). Following isolation and after successful growth of tumor organoids, tumors were sorted on the basis of human EpCAM expression, and all organoids used for testing were from this sorted stock.

#### Culture

Organoids were passaged weekly. Following removal of growth media, Matrigel (Corning, growth factor reduced) domes were mechanically dissociated in TrypLE (Thermo Fisher Scientific) with 10 µmol/L Y27632. Organoids were digested at 37°C for 30–45 minutes, with periodic pipetting to assist in separation. Organoids were collected by centrifugation, washed in DMEM+++, and reseeded 1:5 in 100% Matrigel. Domes were hardened at 37°C for 10 minutes and overlaid with 250 µL media. Media was changed every 3–4 days.

#### Drug Testing

Organoids were seeded as described previously. The following day, media was refreshed with media containing AZD6738. Media was refreshed every 2–3 days for 7 days. Prior to harvest, organoids were imaged on an EVOS brightfield microscope (Thermo Fisher Scientific) at 5X magnification. Next, 125 µL of media was removed from each well and 100 µL of Cell Titer Glo 3D (Promega) was added. Domes were mechanically separated with a pipet and incubated on a rocker for 30–45 minutes at room temperature. A total of 40 µL from each well was moved to a black-walled plate and luminescence was read on a Envision MultiLabel plate reader (Perkin Elmer).

#### Confocal

Confluent organoids were harvested for imaging as follows. Media was removed, and following extensive washing in PBS, Matrigel domes were fixed in 4% PFA in PBS for 60 minutes, with periodic swirling to release whole organoids from Matrigel. A cut p1000 tip was used to transfer whole organoids to a tube, PFA was aspirated, and whole organoids washed several times in PBS. Organoids were permeabilized in perm/block buffer (5% donkey serum, 0.5% Triton X-100 in PBS) for 3 hours at room temperature. Organoids were then incubated with primary antibody (see Table 2 for antibody list) overnight at 4°C. The following day, organoids were washed extensively, and incubated with secondary antibody at room temperature for 3 hours. In the final 10 minutes, 200 µL of an 1 µg/mL DAPI solution was added. Organoids were washed extensively, and moved to chamber-well slides for imaging. Z-stacks were captured on a laser scanning confocal microscope (LSM700, Carl Zeiss) at 8-bit with Plan-Apochromat 20X/1.4NA objective using Zen software (RRID: SCR_013672).

#### Isogenic Organoid Generation

Organoids were infected with lentivirus carrying gRNAs targeting safe-harbor locus *AAVS1*, or *FBXW7*. Organoids were dissociated using TrypLE and incubated with concentrated lentivirus in DMEM+++ media containing 10 µmol/L Y27632 in a spinning centrifuge at 37°C for 6 hours. Following spinoculation, organoids were washed thoroughly in DMEM+++ and plated in matrigel domes. The following day, organoids were selected in 2 µg/mL puromycin for 7 days. Following selection, organoids were expanded in preparation for experiments.

### DNA Combing

To assess DNA replication fork progression in parental and *FBXW7*-knockout HPAF-II cells, 3,000,000 cells of each cell line were cultured in 10 cm^2^ dishes for 24 hours for each biological replicate. Exponentially growing cells were pulsed with 25 mmol/L CldU (Sigma-Aldrich #C6891) for 30 minutes, washed with prewarmed PBS, and then pulsed with 125 mmol/L IdU (Sigma-Aldrich #I7125) for 30 minutes. Cells were then harvested by trypsinization and cast into three 1% low melting point agarose (Bioshop #AGA101) plugs at a density of 5,000,000 cells/mL. The plugs were incubated in 1% *N*­lauroyl sarcosine (Bioshop #SLS002) containing 2 mg/mL Proteinase K (Bioshop #PRK403) at 50°C for 72 hours, with fresh Proteinase K solution being added every 24 hours to digest proteins. Following Proteinase K digestion, the plugs were washed five times for 10 minutes each in TE_50_ buffer (10 mmol/L Tris-HCl pH 7.0, 50 mmol/L EDTA) and a single plug for each sample was melted and processed for DNA combing and immunofluorescence analysis as described previously (17). CldU was detected using rat anti-BrdU (Abcam ab6326, 1:40), IdU was detected using mouse anti-BrdU clone B44 (BD Biosciences #347580, 1:10), and ssDNA was detected using mouse anti-ssDNA clone 16-19 (Millipore MAB3034, 1:40), followed by secondary antibody incubation with Alexa Fluor 488 goat anti-rat IgG (Invitrogen #A-11006, 1:75), Alexa Fluor 546 goat anti-mouse IgG_1_ (Invitrogen #A-21123, 1:50), and Alexa Fluor 647 goat anti-mouse IgG_2a_ (Invitrogen #A-21241, 1:50). Images of more than 200 replication tracks per sample were acquired using a Zeiss AxioImager Z1 fluorescence microscope with a 63× oil-immersion objective lens. DNA replication fork rate was determined by measuring the length of IdU tracks adjacent to CldU tracks using ImageJ (SCR_003070), converting the measured IdU track lengths from pixels to kilobase pairs using a conversion factor based on bacteriophage lambda DNA combing as described previously (17), and dividing by the IdU incubation time (30 minutes) to obtain a measure of replication fork velocity (kbp/minute).

### Clonogenic Growth Assay

HPAF-II wild-type or *FBXW7^−^^/^^−^* cells were infected with lentivirus harboring indicated gRNAs, in the presence of 8 µg/mL of polybrene. The next day, the cells were selected using 2 µg/mL of puromycin for 2 days. Following selection, cells were seeded at 10,000 cells per well for clonogenic growth. The next day, the cells were treated with AZD6738 at indicated concentrations and the media was refreshed every 3–4 days. After 14 days of treatment, cells were washed in PBS and fixed with ice-cold 100% methanol. Cells were stained with 0.25% crystal violet in 25% methanol at room temperature for 15–20 minutes. Plates were imaged on a Bio-Rad Chemidoc MP, destained with 10% acetic acid and absorbance at 595 nm on the Envision Multilabel plate reader (Perkin Elmer) was recorded and plotted.

### Statistical Analysis

All statistical analyses were performed in Graphpad Prism (RRID SCR_002798).

### Research Ethics Statement

Written informed consent was obtained from all patients providing research tissues, and studies were conducted in accordance with the Research Ethics Board at University Health Network, and approved by the University Health Network REB.

### Data Availability Statement

All CRISPR screen data can be founded in Supplementary Data S1, including raw read counts.

## Results

### *FBXW7^−^^/^^−^* Cells Rely on DNA Damage Response Genes and Exhibit High Levels of Replication Stress

**FIGURE 1:**
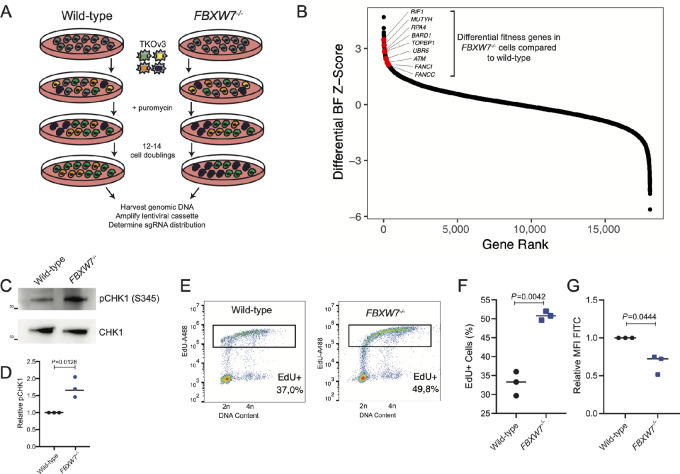
Isogenic genome-wide screen identifies DNA repair genes essential following *FBXW7*-loss. **A,** Schematic of genome-wide CRISPR screens in *FBXW7^−^^/^^−^* and wild-type cells. **B,** Rank order plot summarizing the results of genome-wide screens highlighting the identification of various DNA damage response genes as selectively essential in *FBXW7^−^^/^^−^* HPAF-II cells. BF = Bayes factor, a measure of gene fitness defects upon perturbation. **C,** Western blot analysis of pCHK1 (S345) identifies increased levels of replication stress in *FBXW7^−^^/^^−^* compared with parental wild-type HPAF-II cells. Representative images of three independent replicate experiments. **D,** Quantification of pCHK1 (S345)/CHK1 in HPAF-II wild-type and *FBXW7^−^^/^^−^* cells from C, *n* = 3, Students *t* test. **E,** Dot plots of two-dimensional cell-cycle flow cytometry assessing DNA content and EdU in wild-type and *FBXW7^−^^/^^−^* HPAF-II cells. Representative images of three replicate experiments. **F,** Quantification of percent of cells EdU+ cells in D, *n* = 3, Students *t* test. **G,** Quantification of mean fluorescent intensity (MFI) of EdU+ cells from D, *n* = 3, Students *t* test.

Genome-scale CRISPR fitness screens (11) identified a number of DNA damage response and repair genes as being required for the optimal growth of *FBXW7*-knockout (*FBXW7^−^^/^^−^*) HPAF-II cells when compared to wild-type cells (Fig. 1A and B, Supplementary Data S1). This prompted us to determine whether *FBXW7^−^^/^^−^* cells exhibit higher levels of DNA damage or replication stress. We first assessed the activation of ATR signaling, a key mediator in the cellular response to replication stress. Strikingly, a higher fraction of phosphorylated CHK1, a marker of activated ATR, is present in *FBXW7^−^^/^^−^* cells as measured by phosphorylation at the S345 site (refs. 18, 19; Fig. 1C and D). Cell-cycle profiling following a short EdU pulse demonstrated that *FBXW7^−^^/^^−^* cells have an extended S-phase, as measured by the accumulation of EdU+ S-phase cells, and a slower rate of DNA incorporation as measured by a reduced EdU intensity (Fig. 1E–G; Supplementary Fig. S1A). These results indicate that *FBXW7^−^^/^^−^* cells are experiencing replication stress to a greater degree than the wild-type parental cell line.

**FIGURE 2:**
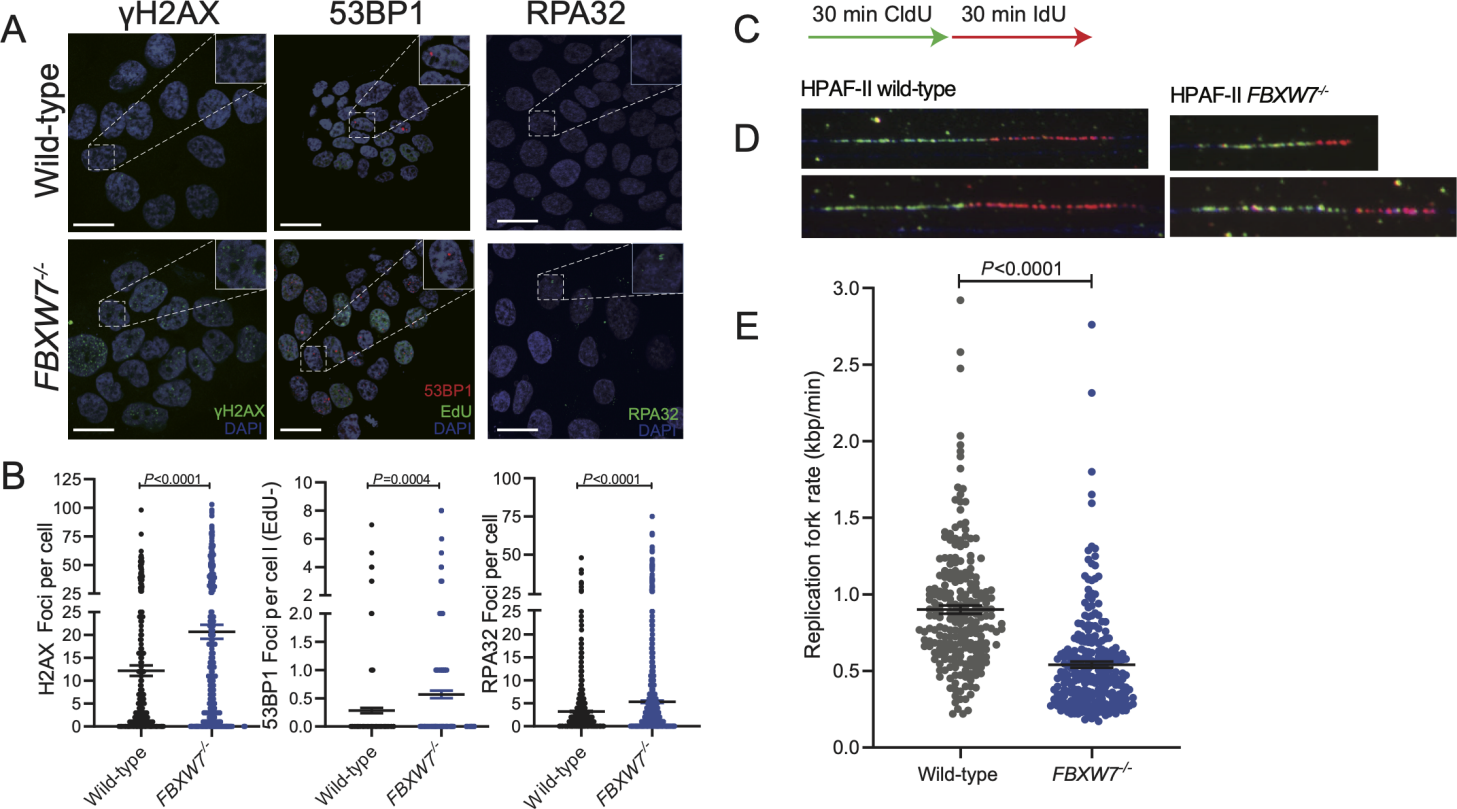
*FBXW7^−^^/^^−^* cells have high levels of replication stress and a reduced replication fork rate. **A,** Immunofluorescence of markers of DNA damage (γH2AX, 53BP1) and replication stress (RPA32) are increased in the *FBXW7^−^^/^^−^* cells compared with wild-type. Representative images of three replicate experiments, scale bar 20 µm. **B,** Quantification of immunofluorescence images, combination of three replicate experiments, mean ± SEM, unpaired *t* test. **C,** Schematic of pulsing strategy used for DNA combing of wild-type and *FBXW7^−^^/^^−^* HPAF-II cells. **D,** Representative images DNA combing experiment highlighting CldU and IdU incorporation. **E,** Quantification of DNA combing experiment in D measuring replication fork rate, unpaired *t* test mean ± SEM *P* < 0.001, minimum of 224 replication tracks were quantified per sample.

We next assessed several key markers of DNA damage and replication stress by immunofluorescence microscopy. An increased number of foci of the DNA damage and replication stress markers γH2AX, 53BP1, and RPA32 were observed in the *FBXW7^−^^/^^−^* cells when compared with parental cells (Fig. 2A and B). The S-phase accumulation observed in *FBXW7^−^^/^^−^* cells (Fig. 1D) could be due either to early entry into S-phase or extended DNA replication. To resolve this question, we performed DNA combing to assess the replication fork rate in wild-type and *FBXW7^−^^/^^−^* cells, and identified heavily impeded fork progression in the *FBXW7^−^^/^^−^* cells (Fig. 2C–E; Supplementary Fig. S1B). Therefore, we conclude that *FBXW7^−^^/^^−^* cells exhibit high levels of replication stress, which we surmise is the cause of growth fitness defects observed upon loss of DNA damage repair genes.

### *FBXW7^−^^/^^−^* Cells are Hypersensitive to ATR Inhibitors

**FIGURE 3:**
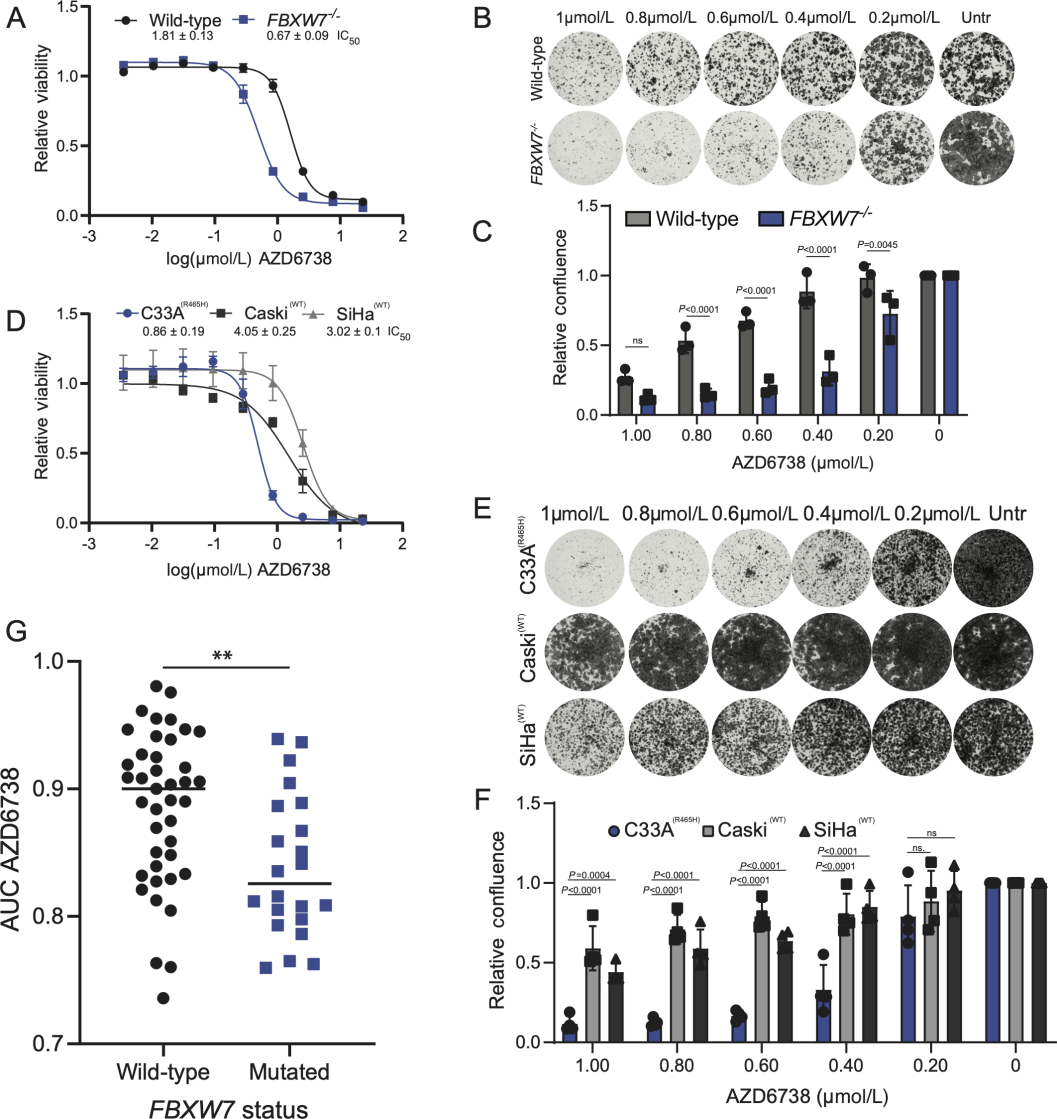
Cells harboring *FBXW7* mutations have enhanced sensitivity to AZD6738. **A,** Dose–response assay using AZD6738 in HPAF-II wild-type and *FBXW7^−^^/^^−^*, representative of three replicates, mean ± SEM. **B,** Clonogenic growth assay in HPAF-II wild-type and *FBXW7^−^^/^^−^*, representative images of three replicates. **C,** Quantification of clonogenic growth assays, *n* = 3, mean ± SEM, two-way ANOVA. **D,** Dose–response assay of cervical cancer cells C33A (*FBXW7^R465H^*), SiHa and Caski (both *FBXW7^WT^*), representative of three replicates, mean ± SEM. **E,** Clonogenic growth assays in a cervical cancer cell line panel, representative images of three replicates. **F,** Quantification of clonogenic growth assays, *n* = 3, mean ± SEM, two-way ANOVA. **G,** AZD6738 sensitivity data from The Cancer Dependency Map (DepMap, Broad Institute) in cancers that harbor high rates of *FBXW7* alterations; cervical cancer, uterine cancer, and colorectal cancer. Mean ± SEM, unpaired *t* test, **, *P* < 0.01.

Considering that a number of DNA damage response genes were uncovered as synthetic lethal with *FBXW7* mutation in the genome-wide CRISPR screen (Fig. 1A), we next wanted to determine whether we could exploit this genetic interaction pharmacologically. ATR is a sensor for ssDNA, and recognizes ssDNA bound by RPA (9). This sensor then goes on to phosphorylate and activate CHK1, a kinase responsible for many downstream activities (19), we demonstrated was activated in *FBXW7^−^^/^^−^* cells (Fig. 1C and D). Phosphorylated CHK1 in turn phosphorylates several substrates, including the key cell-cycle transition regulator CDC25, which leads to cell-cycle arrest as the cells work to correct the DNA damage (20, 21). We therefore selected to test AZD6738, an ATR inhibitor that has a favorable efficacy and safety profile in the clinic (10). We performed a dose–response cell killing assay in wild-type and *FBXW7^−^^/^^−^* cells and observed that *FBXW7^−^^/^^−^* cells are more sensitive to treatment with AZD6738, with an IC_50_ approximately 3-fold lower than that of the parental wild-type cell line (Fig. 3A; Supplementary Fig. S2A). A longer term clonogenic growth assay replicated the results of the dose–response assay (Fig. 3B and C). Next, to confirm that the results were not unique to the HPAF-II genetic background, and indeed were caused by the loss-of-function mutation in *FBXW7*, we assayed a panel of cervical cancer cell lines with diverse genotypes—one of which harbors a loss-of-function mutation (R465H) in *FBXW7* (22). As observed for HPAF-II *FBXW7^−^^/^^−^* cells, the *FBXW7^R465H^*-mutant C33A cervical cancer cell line was 3- to 6-fold more sensitive to AZD6738 treatment than the *FBXW7^WT^*cell lines SiHa and Caski in both dose–response assays and long-term clonogenic growth assays (Fig. 3D–F; Supplementary Fig. S2A). Finally, to understand whether the sensitivity of *FBXW7*-mutant cell lines to AZD6738 is more broadly applicable across cancer types, we mined data generated by the Cancer Dependency map (Broad Institute, Mutation 21Q1, GDSC21917) for three cancer types that harbor a high percentage of *FBXW7* alterations—cervical, colorectal, and uterine cancers, selected for their high rate of alteration frequency identified by The Cancer Genome Atlas Pan-cancer study (13.2%, 15.7%, and 20.5% respectively). For these three cancer types, we identified a significant difference in the sensitivity to AZD6738—with *FBXW7*-mutated cell lines demonstrating higher susceptibility than *FBXW7*-wildtype (Fig. 3G). Furthermore, this significant difference was identified across the entirety of DepMap cell lines (Supplementary Fig. S2B). We conclude that *FBXW7-*mutant cells from multiple cancer types are more sensitive to ATR inhibition, which is broadly applicable to several cancer types, revealing a druggable synthetic lethal interaction between DNA repair genes and *FBXW7* mutations.

Considering the role of CHK1 and WEE1 kinases in mediating the cellular response to replication stress and regulating cell-cycle checkpoints (23), we next assessed whether cells harboring *FBXW7* mutation are more sensitive to inhibition of these kinases downstream of ATR activation. Similar to results following ATR inhibition, *FBXW7^−^^/^^−^* cells were found to be more sensitive to both CHK1 and WEE1 inhibitors, AZD7762 and MK-1775, respectively, than the parental wild-type cell line (Supplementary Fig. S2C and S2E). Furthermore, using the panel of cervical cancer cell lines, we determined that *FBXW7*-mutant cells (C33A) were more susceptible to CHK1 and WEE1 inhibition than *FBXW7* wild-type lines (Supplementary Fig. S2D and S2E). In addition, we tested the novel PKMYT1-inhibitor RP-6306 that was previously characterized to be selectively toxic to cells harboring *CCNE1* amplification or *FBXW7* alterations (24). PKMYT-1 is a WEE1 family kinase that phosphorylates CDK1, slowing entry into mitosis (25). As with the other kinase inhibitors, HPAF-II *FBXW7^−^^/^^−^* cells and C33A cells were both more sensitive to PKMYT1 inhibition than cells carrying wild-type *FBXW7* (Supplementary Fig. S2C–S2E). These data confirm that preventing the activation of the DNA damage response and slowing cell-cycle progression by targeting ATR, CHK1, WEE1, or PKMYT-1 induce preferential killing of *FBXW7*-mutant cells.

### Cervical Cancer Organoids Harboring *FBXW7* Knockout are Hypersensitive to AZD6738

**FIGURE 4:**
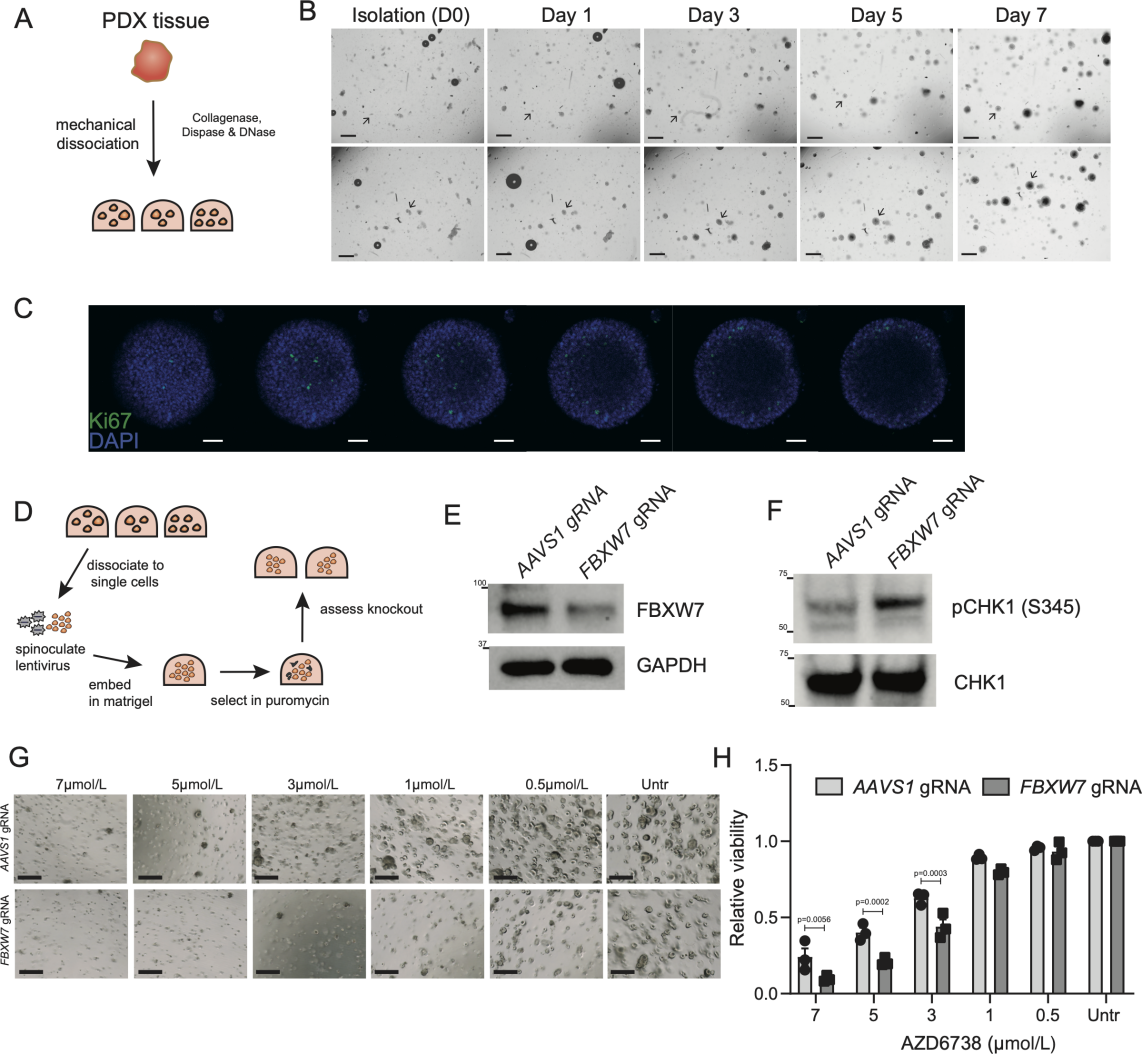
Isogenic *FBXW7*-knockout cervical tumor organoids recapitulate AZD6738 sensitivity. **A,** Schematic of generation of patient-derived cervical tumor organoid cultures. **B,** Imaging of M45 cervical tumor organoid isolation over 7 days, scale bar 200 µm, arrows indicate actively expanding organoids. **C,** Representative Z-stack images of a single organoid stained with DNA stain (DAPI) and marker of proliferation (Ki67), 50 µm scale bar. **D,** Schematic of infection and selection of isogenic cervical tumor organoids. **E,** Representative Western blot analysis of *FBXW7* knockout in isogenic organoids. **F,** Western blot analysis of pCHK1 (S345) in indicated organoids, representative of three independent replicates. **G,** Dose–response assay in organoids treated with AZD6738, over 7 days, scale bars 200 µm. Data are representative of three independent replicates. **H,** Quantification of cell viability relative to untreated cells in three independent replicates of organoid dose–response assay, mean ± SD, two-way ANOVA.

Considering the sensitivity of *FBXW7*-mutant cell lines to AZD6738 (Fig. 2), we next wished to assess whether this mutation-specific sensitivity was conserved in a more complex and biologically relevant tumor model. To achieve this, we generated a cervical cancer organoid line from patient-derived orthotopic xenograft tissue (Fig. 4A and B; refs. 26–28). Tumor organoids grew well following isolation exhibiting a cystic morphology and areas of proliferation as assessed through Z-stack confocal imaging and Ki67 staining (Fig. 4C). We then generated isogenic models of this organoid line (M45) by infecting organoids with lentivirus harboring Cas9 and a gRNA targeting a control region (*AAVS1)* or *FBXW7* (Fig. 4D). Knockout efficiency was assessed by Western blotting (Fig. 4E) and sequencing (Supplementary Fig. S3A). Next, we asked whether high levels of replication stress were present in the *FBXW7^−^^/^^−^* organoids by assessing pCHK1 (S345) levels. Indeed, the *FBXW7^−^^/^^−^* organoids had an increased activation of ATR in comparison to the parental organoids (Fig. 4F; Supplementary Fig. S3B). We next performed dose–response assays using the ATR inhibitor AZD6738. Through imaging and viability readouts, our results demonstrated that *FBXW7^−^^/^^−^* organoids are more sensitive to AZD6738 treatment when compared with the parental organoids (Fig. 4G and H).

### Control of S–G_2_ Transition Mediates Response to ATR Inhibitor in *FBXW7^−^^/^^−^* Cells

**FIGURE 5:**
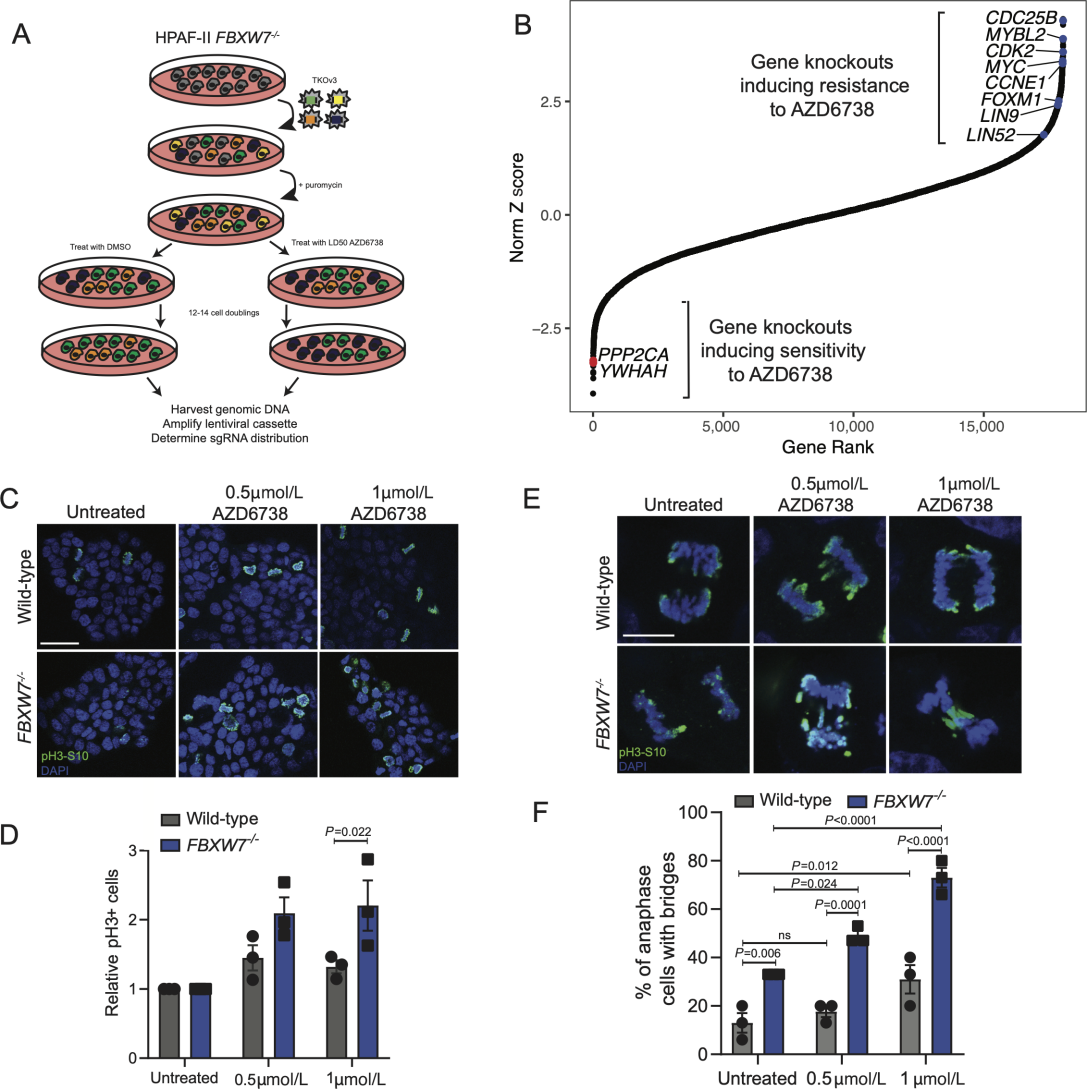
Loss of control at S–G_2_ transition induces sensitivity to ATR inhibitor. **A,** Schematic representation of chemogenomic CRISPR screen in HPAF-II *FBXW7^−^^/^^−^* cells using an LD50 dose of AZD6738. **B,** Results of chemogenomic screen ranked by Z-score generated through DrugZ. **C,** Immunofluorescence of HPAF-II wild-type and *FBXW7^−^^/^^−^* cells treated or untreated with AZD6738 and stained for phosphorylated histone H3. Representative images of three independent replicates, scale bar is 50 µm. **D,** Quantification of **C**, mean ± SEM, two-way ANOVA. **E,** Immunofluorescence of HPAF-II wild-type and *FBXW7^−^^/^^−^* cells during anaphase, representative images of three independent replicates, scale bar is 10 µm. **F,** Quantification of E, 15 cells per replicate per treatment were imaged. Mean ± SEM, two-way ANOVA.

To understand the mechanisms underlying the increased AZD6738 sensitivity in the HPAF-II *FBXW7^−^^/^^−^* cell line, we performed a CRISPR chemogenomic screen (Fig. 5A). DrugZ analysis identified gene knockouts that drive resistance to AZD6738—which included both *CCNE1* and *CDK2*—gatekeepers of the G_1_–S cell-cycle phase transition (Fig. 5B). The G_2_–M phosphatase *CDC25B* was also identified (Fig. 5B). During the cell cycle, CDC25B is tasked with dephosphorylating CDK2, driving the cells to enter mitosis (29). Interestingly, multiple members of a large protein complex tasked with initiating expression of early G_2_ genes (*MYBL2, FOXM1, LIN9, LIN52*) required for transition out of S-phase were also identified as knockouts that drive resistance to AZD6738, further validating the hypothesis that slowing the transition out of S-phase reduces the toxicity of AZD6738 (30, 31). These identified genes make up components of the DREAM complex, which has recently been reported to act as a transcriptional repressor of DNA damage repair, supporting our results that loss of DREAM components reduces sensitivity to ATR inhibition by slowing exit from S-phase (32).

Conversely, *PPP2CA,* the catalytic subunit of protein phosphatase 2C, was identified as sensitizing cells to AZD6738, as was *YWHAH* that encodes 14-3-3 eta (Fig. 5B; Supplementary Fig. S4A, S4B, and S4G). Both PP2A and 14-3-3 have been described as negative regulators of CDC25B through dephosphorylation and cytoplasmic sequestration (33). Individual knockout of screen hits validated the increased sensitivity to AZD6738 (gRNA-*PP2CA*) or acquisition of AZD6738 resistance (gRNA-*CDC25B* or *CCNE1*; Supplementary Fig. S4A–S4I). Variability in both cell growth and gRNA editing efficiency in the *CDC25B* knockouts is observed, and further testing of additional gRNAs with both gene-level and protein-level editing efficiency testing is an important next step to confirm these findings. The screen results clearly indicate that control of cell-cycle progression is essential in mediating response to ATR inhibition, and slowing G_2_ entry can induce resistance to inhibiting ATR.

### AZD6738 Induces an Accelerated S-phase and Mitotic Catastrophe

Considering the strong evidence that slowing down S-phase exit provides resistance to AZD6738 from our genome-wide chemogenomic screen (Fig. 5B), combined with previous work describing the ATR's role in mediating S–G_2_-phase transition (8, 9), we next asked whether AZD6738 treatment altered S-phase timing in HPAF-II wild-type and *FBXW7^−^^/^^−^* cells. First, through immunofluorescence detection of phosphorylated histone H3, we identified that ATR inhibition in both wild-type and *FBXW7^−^^/^^−^* HPAF-II cells induced accumulation of phosphorylated histone H3 with a greater accumulation found in *FBXW7^−^^/^^−^* cells (Fig. 5C and D). This finding is in line with previously observed effects of ATR inhibition (8, 9) and suggests that control of the S–G_2_ transition is altered when treated with the inhibitor. Use of the PIP-FUCCI cell-cycle reporter (11, 34) confirmed these findings, demonstrating enhanced accumulation of G_2_–M in the *FBXW7^−^^/^^−^* background following treatment with AZD6738, and a shortened S-phase (Supplementary Fig. S4J–S4M). Considering the high levels of replication stress in the *FBXW7^−^^/^^−^* cell line, we hypothesized that a shortened S-phase may cause under-replicated or damaged DNA to enter G_2_–M-phase, which could lead to aberrant mitosis or mitotic catastrophe. Following treatment with two doses of ATR inhibitor for 48 hours, we counted cells undergoing anaphase and assessed whether anaphase bridges were forming. Consistent with our hypothesis, anaphase bridges were detected at a higher rate and at lower doses of AZD6738 in the *FBXW7^−^^/^^−^* cell line when compared with wild-type cells (Fig. 5E and F). Importantly, further study into whether cells are arrested in mitosis, or have exited S-phase early, is important to understand exactly how this sensitivity is occurring. These results confirm that high levels of replication stress in *FBXW7*-mutant cells confer heighten sensitivity to chemotherapeutic agents that interrupt the S–G_2_ transition, such as ATR inhibitors, through mitotic catastrophe.

## Discussion

It is perhaps unsurprising that cells harboring loss-of-function mutations in *FBXW7* cells have an enhanced requirement for DNA replication stress response genes. *FBXW7* is a powerful tumor suppressor gene, and it functionally regulates the abundance of two proto-oncogenes; MYC and cyclin E. Accumulation of these proto-oncogenes is likely contributing to high levels of replication stress observed in *FBXW7^−^^/^^−^* cells. Indeed, targeting *CCNE1*-high cancers with inhibitors of the ATR signaling pathway is a well-studied strategy (24, 35–37). Results from our chemogenomic screen demonstrate that cyclin E loss does induce some resistance to AZD6738, as does *MYC* loss (Fig. 5B) suggesting that these two oncogenes are important determinants mediating the cellular response to ATR inhibition. These findings also suggest that regulation at the G_1_–S-phase is critical to determine sensitivity to ATR inhibition. Indeed, several groups have shown that ATR pathway inhibition is more efficacious in p53-mutated cells (38–41), which also exhibit a weakened G_1_–S checkpoint. It is clear that loss of G_1_ checkpoint control enhances reliance on the G_2_–M checkpoint to ensure cells with DNA damage or under-replicated DNA do not enter mitosis.

High levels of replication stress in *FBXW7^−^^/^^−^* cells cause a delayed progression through S-phase. Our data show that *FBXW7^−^^/^^−^* cells are indeed accumulating in S-phase (Fig. 1E and F; Supplementary Fig. S1B), that replication is progressing at a slower rate compared with wild-type (Fig. 2C–E; Supplementary Fig. S1A), and *FBXW7^−^^/^^−^* cells have an elongated S-phase in comparison to wild-type (Supplementary Fig. S4I). This evidence supports the conclusion that *FBXW7^−^^/^^−^* cells are undergoing replication stress and that an extended S-phase is sufficient to replicate the genome under these conditions without affecting overall cell fitness. However, when treated with inhibitors that block S-phase extension, that is, targeting ATR, CHK1 or WEE1, *FBXW7^−^^/^^−^* cells exhibit increased sensitivity than wild-type cells. Looking forward, it is important to understand whether the G_2_-phase of the cell cycle is being affected in these cell types, both with and without treatment.

Control of the G_2_–M checkpoint is absolutely essential for *FBXW7^−^^/^^−^* cells. Using inhibitors of ATR, CHK1, WEE1, and PKMYT1 we have demonstrated that this requirement is robust and can be targeted at various stages of the G_2_–M checkpoint. Both CHK1 and WEE1 inhibitors have a similar mechanism of action in inducing a forced mitotic entry (42–44), and the status of the G_1_–S checkpoint in cells can predict responses to WEE1 inhibition (45). Chemogenomic screening using the CHK1 inhibitor prexasertib uncovered a similar set of resistance genes as we describe here, highlighting the importance of blocking mitotic progression to promote resistance to both CHK1 and ATR inhibitors (31, 43). In addition, a resistance-based screen using PKMYT1 inhibitor RP-6306 also identified a similar subset of genes providing resistance, further supporting that mitotic progression is required for the efficacy of inhibitors targeting various components of the ATR signaling pathway (24). Further work *in vivo* to assess the viability of these inhibitors in this cancer context is needed, as well as an assessment of toxicity in nonmalignant cell line models. Interestingly, despite the selectivity these inhibitors demonstrate between wild-type and *FBXW7^−^^/^^−^* cells, there are no major differences in the gene essentiality of *PKYMT1*, *WEE1*, and *CHEK1*, with a modest increase in the requirement of *ATR* in *FBXW7^−^^/^^−^* cells. Though a large difference in the essentiality of *ATM* in wild-type versus *FBXW7^−^^/^^−^* cells, in both cell lines *ATM* remained a non-essential gene, with a Bayes factor below zero.

Our work highlights the use of a well-studied inhibitor of the ATR kinase for use in cancers harboring loss-of-function mutations in *FBXW7*, which expands the pool of patients who could respond well to this treatment outside of those with mutations in core DNA damage response genes who are currently being included in clinical trials. Considering the positive early data on the use of AZD6738 in the clinic, stratification of patients based on *FBXW7* mutational status may boost clinical success especially considering the high prevalence of *FBXW7* mutation across cancers.

## Supplementary Material

Supplementary Data 1 Raw read counts and bayes factors for CRISPR screen

Figure S1 Supplementary figure S1 shows flow cytometry gating strategy and additional DNA combing data

Figure S2 Supplementary figure S2 shows dose responses assays and IC50 calculations for ATR pathway inhibitors

Figure S3 Supplementary figure S3 shows CRISPR editing analysis and quantification of organoid assays

Figure S4 Supplementary figure S4 shows the validation of the AZD6738 chemogenomic screen and quantification of cell cycle assays following treatment with AZD6738
