# Supplementary material for: FBXW7-loss Sensitizes Cells to ATR Inhibition Through Induced Mitotic Catastrophe
Source: Cancer Res Commun. 2023 Dec 21;3(12):2596–607. doi: 10.1158/2767-9764.CRC-23-0306 (PMC10734389; doi:10.1158/2767-9764.CRC-23-0306)
Supplement: Figure S1 — Supplementary figure S1 shows flow cytometry gating strategy and additional DNA combing data [file crc-23-0306-s02.pdf]

Figure S1

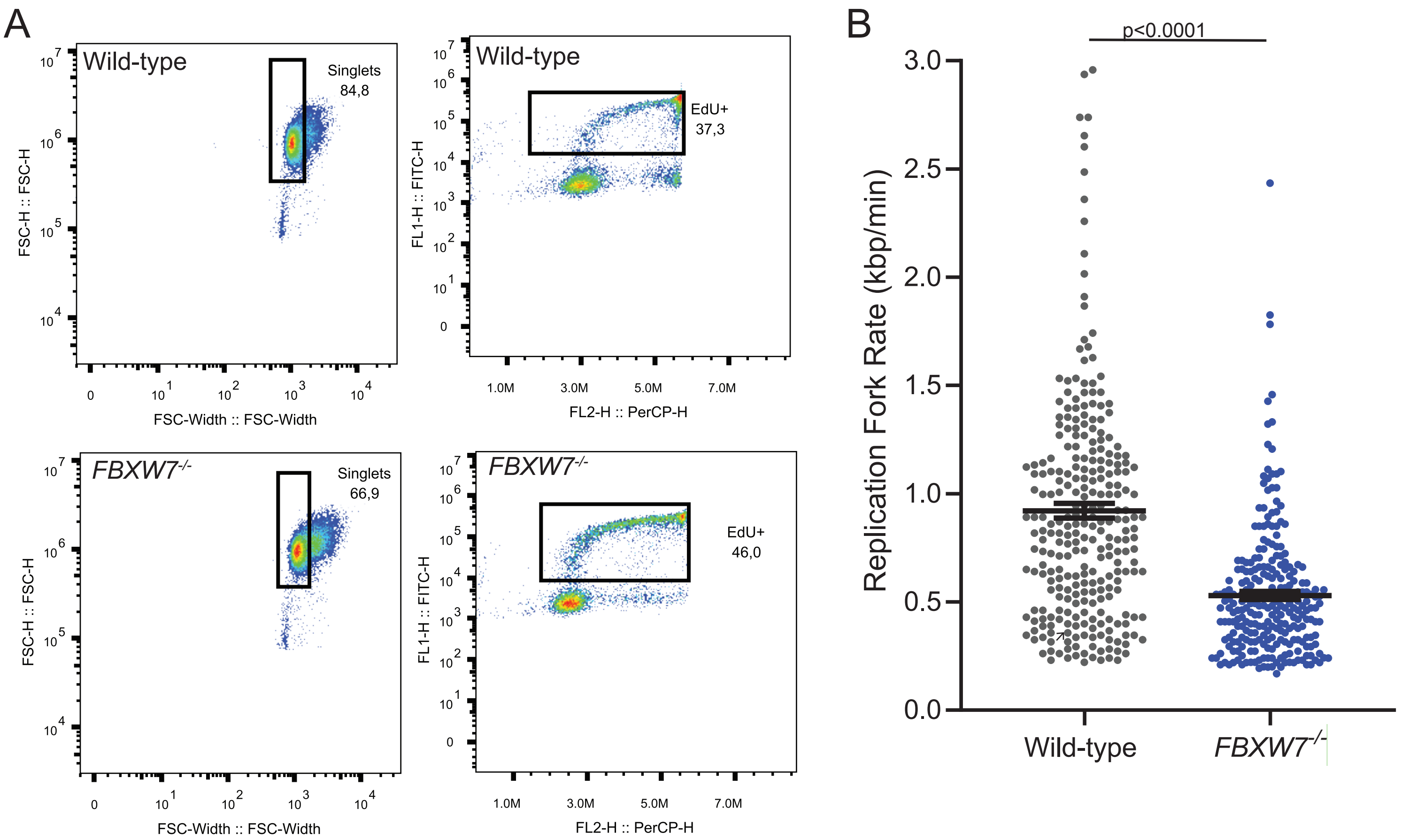

Figure S1. Flow cytometry gating strategy and additional DNA combing data
A) Representative gating strategy for HPAF-II wild-type and FBXW7-/- cells for EdU incorporation assay. B) Second replicate of DNA combing assay in HPAF-II wild-type and FBXW7-/- cells, unpaired t-test.
