# Supplementary material for: FBXW7-loss Sensitizes Cells to ATR Inhibition Through Induced Mitotic Catastrophe
Source: Cancer Res Commun. 2023 Dec 21;3(12):2596–607. doi: 10.1158/2767-9764.CRC-23-0306 (PMC10734389; doi:10.1158/2767-9764.CRC-23-0306)
Supplement: Figure S2 — Supplementary figure S2 shows dose responses assays and IC50 calculations for ATR pathway inhibitors [file crc-23-0306-s03.pdf]

Figure S2

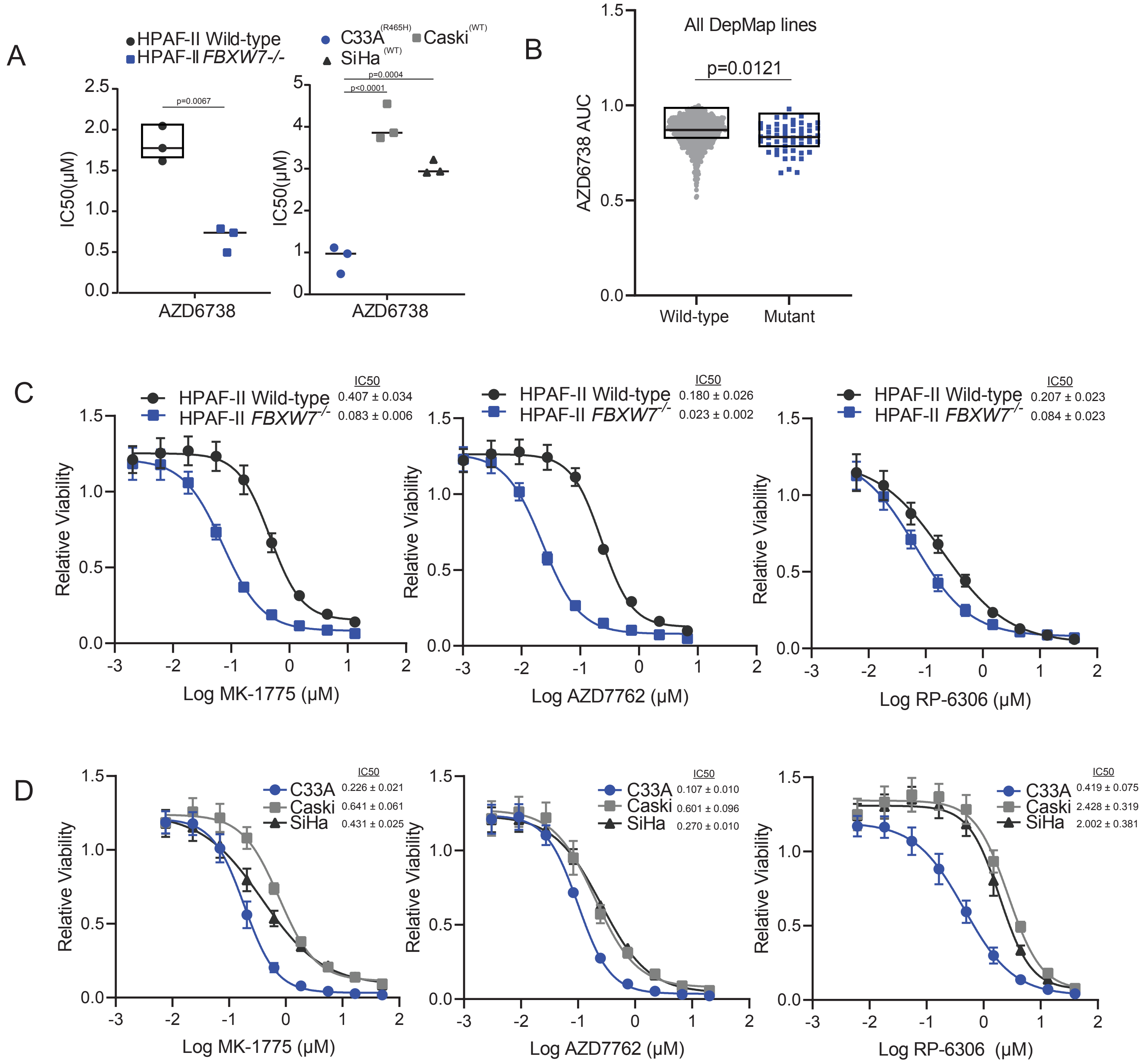

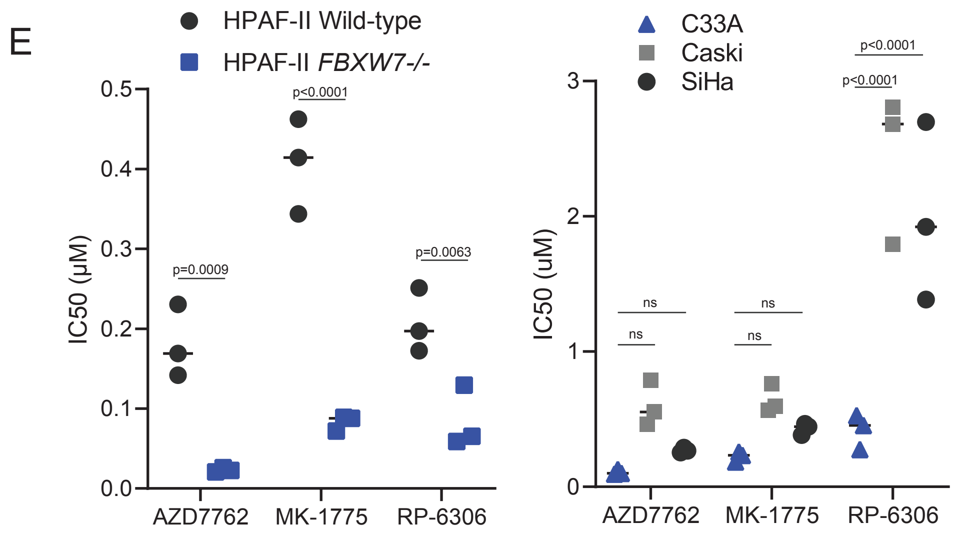

Figure S2. Dose responses assays and IC50 calculations for ATR pathway inhibitors
A) IC50 plot of HPAF-II wild-type and FBXW7-/- cells, and cervical cell line panel treated with AZD6738, mean of three independent replicates, paired t-test and one-way ANOVA respectively. B) Plot of AUC values for AZD6738 from DepMap of all cell lines with and without FBXW7-mutation, t-test. C) Dose-response assay with MK-1775, AZD6738, RP-6306 in HPAF-II wild-type and FBXW7-/- cells, representative of three replicates, mean ± SEM. D) Dose-response assays with MK-1775, AZD7762, RP-6306 in cervical cancer cell line panel (C33A harbours a FBXW7R465H mutation), representative of three replicates, mean ± SEM. E) IC50 plot of HPAF-II wild-type and FBXW7-/- cells, and cervical cell line panel treated with MK-1775, AZD7762, RP-6306, mean of three independent replicates, 2-way ANOVA.
