# Supplementary material for: FBXW7-loss Sensitizes Cells to ATR Inhibition Through Induced Mitotic Catastrophe
Source: Cancer Res Commun. 2023 Dec 21;3(12):2596–607. doi: 10.1158/2767-9764.CRC-23-0306 (PMC10734389; doi:10.1158/2767-9764.CRC-23-0306)
Supplement: Figure S3 — Supplementary figure S3 shows CRISPR editing analysis and quantification of organoid assays [file crc-23-0306-s04.pdf]

Figure S3

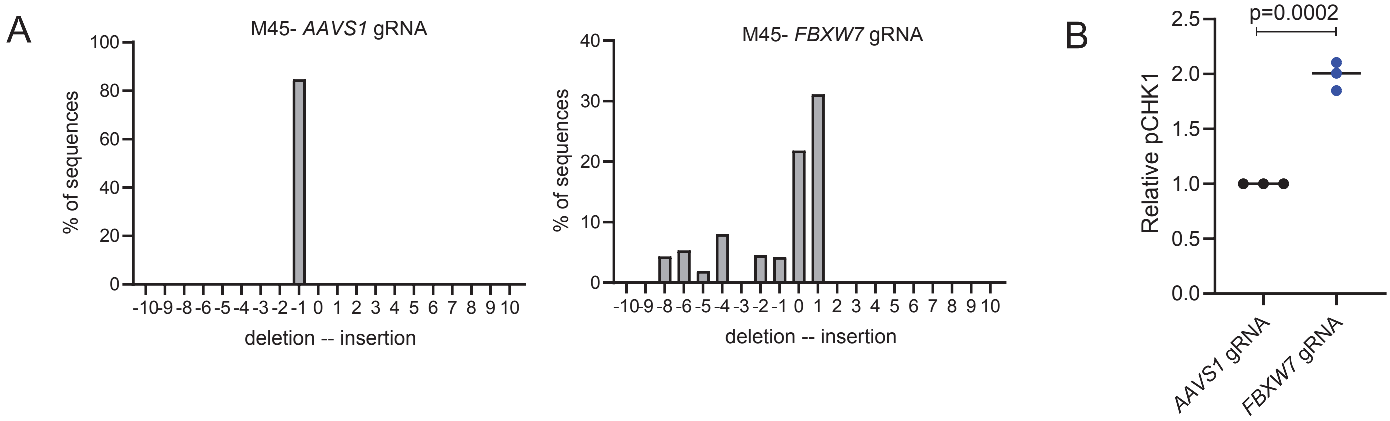


Figure S3. CRISPR editing analysis and quantification of organoid assays
A) TIDE analysis of organoids infected with indicated gRNAs. B) Quantification of pCHK1/CHK1 in isogenic organoids, n=3, students t-test.
