# Supplementary material for: FBXW7-loss Sensitizes Cells to ATR Inhibition Through Induced Mitotic Catastrophe
Source: Cancer Res Commun. 2023 Dec 21;3(12):2596–607. doi: 10.1158/2767-9764.CRC-23-0306 (PMC10734389; doi:10.1158/2767-9764.CRC-23-0306)
Supplement: Figure S4 — Supplementary figure S4 shows the validation of the AZD6738 chemogenomic screen and quantification of cell cycle assays following treatment with AZD6738 [file crc-23-0306-s05.pdf]

Figure S4

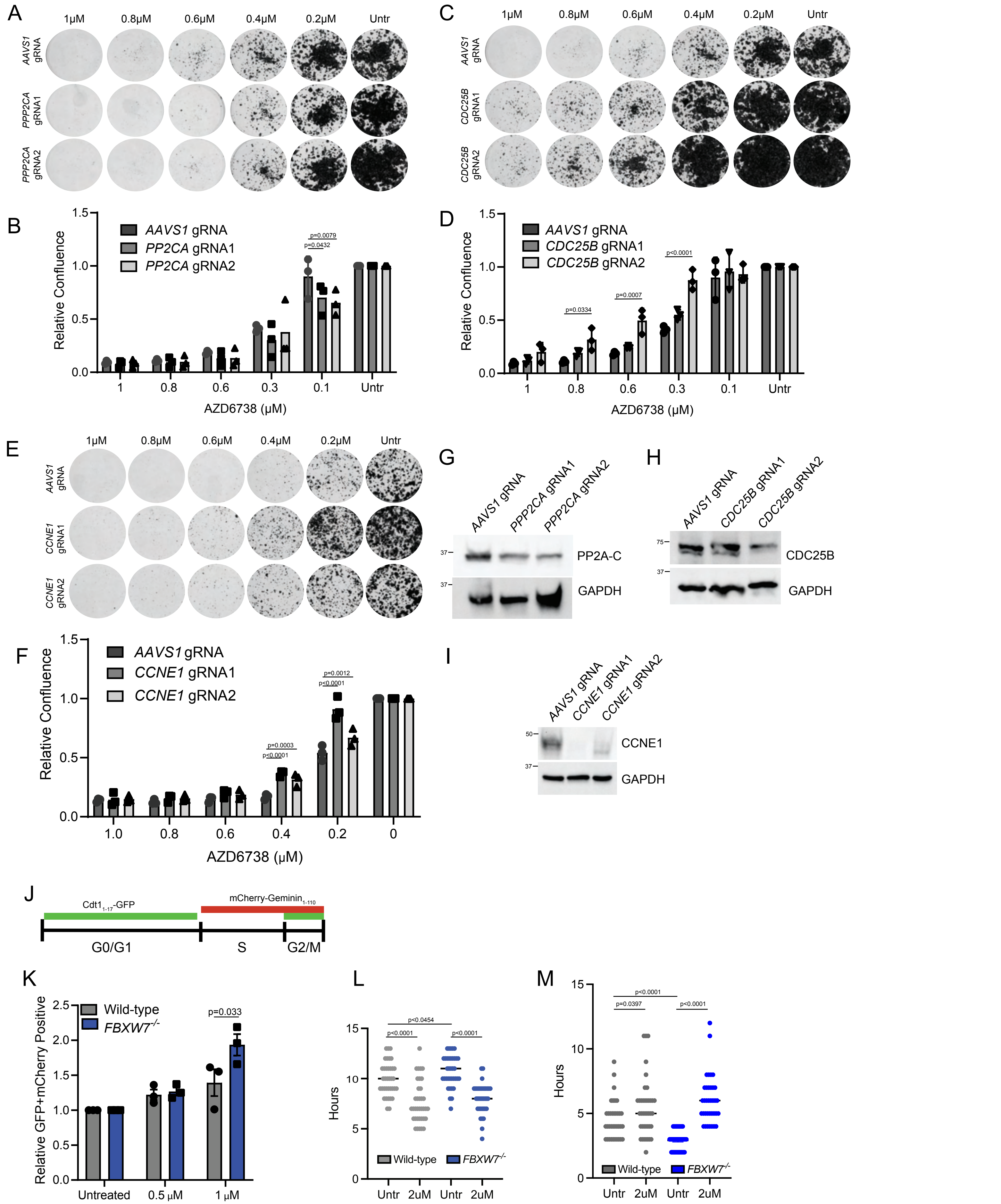

Figure S4. Validation of chemogenomic screen and quantification of cell cycle assays following treatment with AZD6738

A) Clonogenic growth assay in HPAF-II FBXW7-/- cells, in the presence of AZD6738 with two independent knockouts of PPP2CA demonstrating enhanced sensitivity to AZD6738. Representative images of three independent replicates. B) Quantification of clonogenic growth assay in A, three independent replicates, mean ± SEM, 2-way ANOVA. C) Clonogenic growth assay in HPAF-II FBXW7-/- cells, in the presence of AZD6738 with two independent knockouts of CDC25B demonstrating enhanced sensitivity to AZD6738. Representative images of three independent replicates. D) Quantification of clonogenic growth assay in C, three independent replicates, mean ± SEM, 2-way ANOVA. E) Clonogenic growth assay in HPAF-II FBXW7-/- cells, in the presence of AZD6738 with two independent knockouts of CCNE1 demonstrating resistance to AZD6738. Representative images of three independent replicates. F) Quantification of clonogenic growth assay in A, three independent replicates, mean ± SEM, 2-way ANOVA. G) Western blot of gRNA editing efficiency against PP2A-C. H) Western blot of gRNA editing efficiency against CDC25B. I) Western blot of gRNA editing efficiency against CCNE1. J) Schematic representation of PIP-FUCCI reporter. K) G2/M accumulation (GFP/mCherry double-positive) of HPAF-II wild-type and FBXW7-/- cells following 48h treatment of AZD6738, three independent replicates, mean ± SEM, 2-way ANOVA. L) S-phase length in PIP-FUCCI reporter cells as measured by time mCherry positive, 12 cells counted per replicate, three independent replicates pooled, unpaired t-test. M) G2/M phase length in PIP-FUCCI reporter cells as measured by time mCherry/GFP double-positive, 12 cells counted per replicate, three independent replicates pooled, unpaired t-test.
